# A non-classic form of McCune Albright syndrome with different presentations and review of the literatures

**DOI:** 10.22088/cjim.12.0.401

**Published:** 2021

**Authors:** Alireza Navabazam, Fatemeh Owlia, Mohammad Hassan Akhavan Karbassi, Roqayeh Hakimian

**Affiliations:** 1 Department of Oral and Maxillofacial Surgery, Shahid Sadoughi University of Medical Sciences, Yazd, Iran; 2 Department of Oral Medicine, Shahid Sadoughi University of Medical Sciences, Yazd, Iran; 3 Shahid Sadoughi University of Medical Sciences, Yazd, Iran

**Keywords:** Cafe-au-Lait spots, Fibrous dysplasia, Polyostotic, Puberty, Precocious

## Abstract

**Background:**

McCune Albright syndrome (MAS) is a rare heterogeneous clinical syndrome without any predilection for ethnic group. Classic form includes triad of fibrous dysplasia, café au late spots and autonomous hyper function of one or more endocrine pathways.

**Case Report:**

We report the case of an 18-year old girl with non-classic form of MAS .New aspect of this case report attributed to multiple sebaceous adenoma.

**Conclusion:**

The new finding of our case of MAS was not reported before. Periodic follow-up with different radiologic and laboratory tests should be considered after suspicion to MAS.

The classic form of McCune Albright syndrome (MAS) includes triad of fibrous dysplasia, café au late spots and autonomous hyper function of one or more endocrine pathways (1). Fibrous dysplasia (monostotic or poly ostotic) in face and extremities are the bone involvement of this syndrome. Endocrinopathy regarding the location of the mutation could be presented in different patterns like gonadotropin independent precocious puberty, acromegaly or hyper thyroidism in most cases (2). New aspect of this case report attributed to multiple sebaceous adenoma. This finding was not reported in MAS, before. Post-zygotic somatic mutation in the gene GNAS 1 is the main etiology (3). Therefore altered non classic form of MAS should be expected, if late mutation has occurred (2). 

## Case Presentation

**Figure 1:**
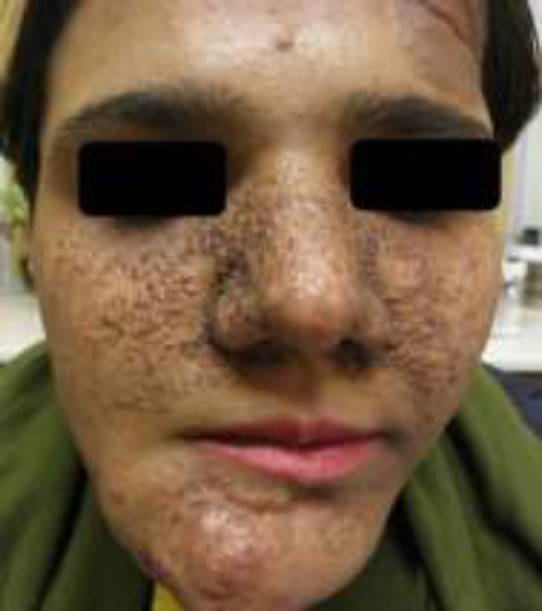
Multiple sebaceous adenoma and asymmetry on the face

**Figure 2:**
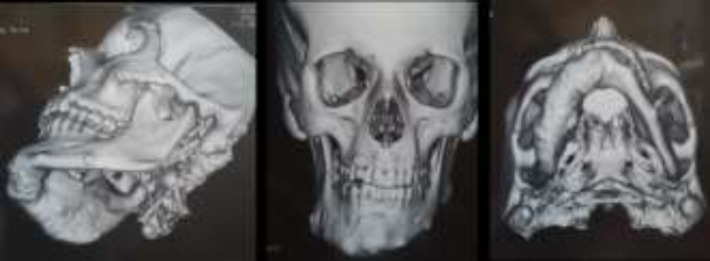
Poly-ostotic fibrous dysplasia of skull

An 18-year-old female referred to oral medicine department, complaining facial asymmetry. It had increased gradually 5 years ago. She was on carbamazepine since she was 4 years old, because of childhood seizure. Diffused papulo-nodular lesions observed on her face (figure 1). Review of her system showed facial asymmetry was related to poly-ostotic fibrous dysplasia after 3dimentional computed tomography (CT) (figure 2). It did not show any document for long bone involvement. She had two skin macules on the right side of her back that were compatible with café au late with ragged borders. Her right ear hearing was completely lost at age 15. There is no similar case in her family and her seven siblings had not any similar problems. Vision test was normal. She had normal puberty process from what she remembered. Diagnosis of sebaceous adenoma was confirmed after excisional biopsy of facial lesions. Café au late skin spots were in the right side with irregular borders that is not compatible with neurofibromatosis type 1(4).

Clinical documents usually is sufficient to diagnosis of MAS, while making diagnosis of fibrous dysplasia in most cases achieved with plain radiographs and confirmed after microscopic evaluation (5). As a stereotype "Chinese letter" appearance in histopathology and ground glass pattern in radiography led to diagnosis of fibrous dysplasia (6).

## Discussion

Multiple wart-like sebaceous adenoma on face and oral cavity, seizure, mental retardation, glial proliferation and neural deformity in CNS. Putting aside sebaceous adenoma, no similarity was observed. Multiple type of Café au late macule, in spite of solitary form-point to genetic disorders like neurofibromatosis type 1(NF1), Mccune Albright and Noonan syndromes. In the absence of inclusive criteria of NF1 are multiple neurofibroma, Crow^e,s^ sign and Lisch nodules in this case, NF1was ruled out (7).

**Figure 3:**
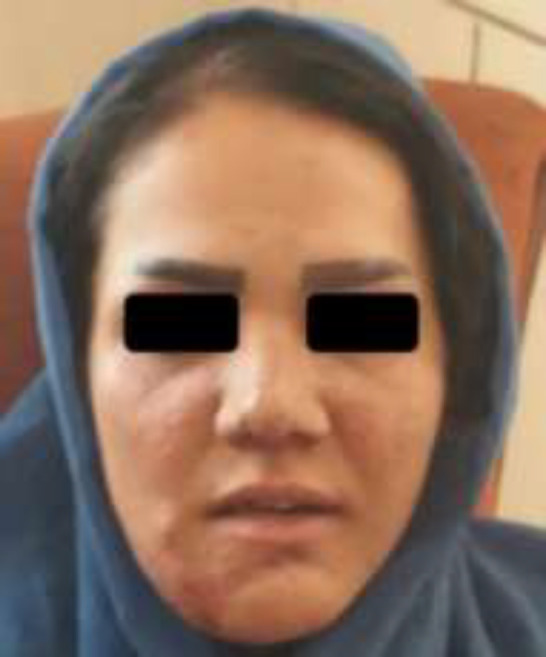
Facial feature of the patient after aesthetic surgery and 5 years follow- up

Incidence of café au late spots in MAS reported 60 to 95% (8). It is worthy to be mentioned that usually signs and symptoms of MAS occurred unilaterally. Our case was not an exception, fibrous dysplasia and hearing loss were in the right side of the body. Café au late spots could be perceived as an alarm sign to diagnose MAS. They usually appear either in time of birth or shortly after that (9). Estimation of occurrence of café au late spots in MAS ranged from 53.1 to 92.5% (10). Because of the personalized pattern of disorder in patients, there are not specific medications to treat bone involvement. Drugs like alendronate relatively are palliative (11). Therefore she was referred to plastic surgeon for shaving of the bone and aesthetic surgery. She underwent aesthetic surgery seven times (figure 3). Now she is 25 years old and her seizure is in control with carbamazepine (200mg daily) and clonazepam (1mg daily).

In spite of other cases which have been reported by pediatrics or endocrinologist, this case considering fibrous dysplasia of face as a diagnostic mile stone, has been reported by oral medicine specialist and oro-maxillo-facial surgeon. This is according to the late onset of some endocrinal disorders of MAS during life. Periodic follow-up with different radiologic and laboratory tests should be considered after suspicion to MAS.
